# Single-photon imaging in complementary metal oxide semiconductor processes

**DOI:** 10.1098/rsta.2013.0100

**Published:** 2014-03-28

**Authors:** E. Charbon

**Affiliations:** Department of Microelectronics, Delft University of Technology, Mekelweg 4, 2628 CD Delft, The Netherlands

**Keywords:** single-photon avalanche diode, avalanche photodiode, complementary metal oxide semiconductor

## Abstract

This paper describes the basics of single-photon counting in complementary metal oxide semiconductors, through single-photon avalanche diodes (SPADs), and the making of miniaturized pixels with photon-counting capability based on SPADs. Some applications, which may take advantage of SPAD image sensors, are outlined, such as fluorescence-based microscopy, three-dimensional time-of-flight imaging and biomedical imaging, to name just a few. The paper focuses on architectures that are best suited to those applications and the trade-offs they generate. In this context, architectures are described that efficiently collect the output of single pixels when designed in large arrays. Off-chip readout circuit requirements are described for a variety of applications in physics, medicine and the life sciences. Owing to the dynamic nature of SPADs, designs featuring a large number of SPADs require careful analysis of the target application for an optimal use of silicon real estate and of limited readout bandwidth. The paper also describes the main trade-offs involved in architecting such chips and the solutions adopted with focus on scalability and miniaturization.

## 1. Introduction

**Figure 1.**
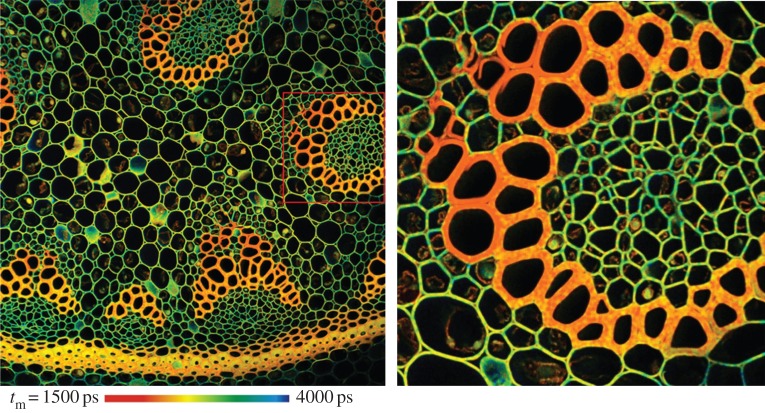
FLIM images obtained through TCSPC on a scanned confocal microscope, courtesy of Dr Wolfgang Becker. The colour-coded image shows the lifetime map of a multi-fluorophore-stained sample to evidence certain cellular membrane details.

Single-photon sensors have existed for decades implemented in various technologies and operating in a range of environmental conditions, at cryogenic temperatures, in high magnetic fields, or in high levels of radioactivity. In this paper, we focus on solid-state single-photon sensors that have existed since the early days of transistors. Among the most promising such sensors, complementary metal oxide semiconductor (CMOS) avalanche photodiodes (APDs) have emerged as the most versatile and easy to use [1]. CMOS APD-based sensors have many applications, wherever time resolution and extreme photon flux conditions exist. A technique often used in biological research, known as fluorescence lifetime imaging microscopy (FLIM), consists of identifying families of molecules based on the mean time required by the molecule to go from excited to ground state. This, known as lifetime, generally ranges from a few hundred picoseconds to several microseconds. Often, selectivity requires that the discrimination of lifetimes be of the order of 100 ps. A useful technique to achieve this level of accuracy is known as time-correlated single-photon counting (TCSPC) [2], which, in combination with specific fluorescent molecules, or fluorophores, enables the tagging of certain areas of interest, as shown for example in figure 1. Figure 1 was obtained on a confocal microscope by exposing the sample to very short (typically hundreds of femtoseconds to several picoseconds) laser pulses that cause its molecules to migrate from the ground state to an excited state. The return to ground state releases photons at a random time, which can be measured a few nanoseconds after the excitation. In order to improve the statistics of the measurement, the experiment is repeated several thousand times, and the expected excitation time is computed. The colour code in the figure relates to the measured molecular lifetime of the fluorophores used to stain the sample.

Figure 1 has a resolution of 2048×2048 pixels and 256 time bins spanning lifetimes from 1500 to 4000 ps. The fluorophores used in this case were Alexa 488 and Mitotracker.

**Figure 2.**
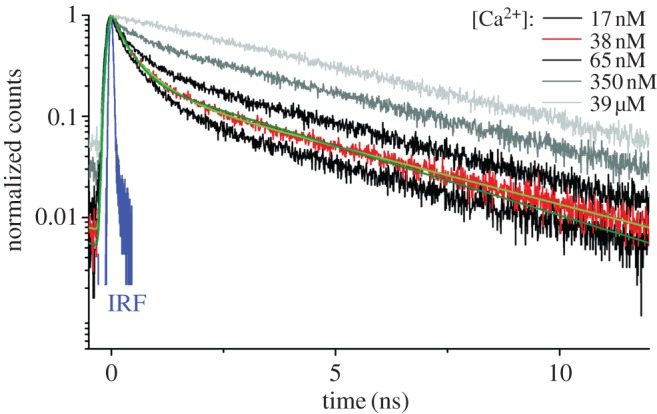
Histograms of the response of OGB-1 molecules to repeated excitation in the presence of Ca^2+^ ions at various concentrations [3]. In this case, TCSPC was used to reconstruct the lifetime of the fluorophore OGB-1 as a function of calcium concentration to monitor neuron activity non-destructively. The figure also shows the response of the optical set-up in the absence of fluorophore to characterize its instrument response function (IRF). (Online version in colour.)

FLIM can also be used to non-destructively image certain intracellular dynamics, such as calcium transport and exchange within natural neural networks. Figure 2 shows a typical photon response of a sample with high-affinity non-ratiometric Ca^2+^ indicator Oregon Green BAPTA-1 (OGB-1) in a solution of calcium ions of various concentrations [3]. Using TCSPC with an overall impulse response function of 79 ps, the fluorescence dynamics of OGB-1 was found to follow a triple exponential decay, thus providing an accurate model of the relation between concentration and decay parameters.

**Figure 3.**
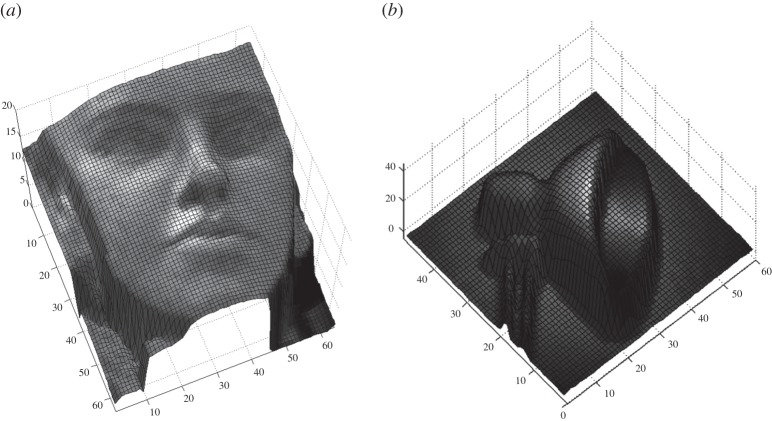
Example of three-dimensional reconstruction obtained using two different optical TOF systems: (*a*) TCSPC [4,5] and (*b*) single-photon synchronous detection [8,9]. Both systems detect the returning photons and their time of arrival to derive the overall TOF and thus reconstruct the distance from the camera to the target.

Time-resolved single-photon imaging can find other applications. One of them is time-of-flight (TOF) imaging, for example. A camera where each pixel can acquire TOF measurements of the environment could be used to reconstruct the real world in three dimensions, while, at the same time, determining the absolute distance of approaching objects using an active, pulsed illumination system. Such a system would act essentially in TCSPC mode, where lifetime is unimportant, whereas the actual position of the peak response is used to determine the TOF and thus the distance of the reflection [4–9]. Figure 3 shows an example of reconstructed object using TOF and a sensor operating in two different modes. Three-dimensional vision based on TOF is becoming increasingly important in emerging fields such as conservation, consumer and industrial robotics, gaming and autonomous or vision-assisted driving.

**Figure 4.**
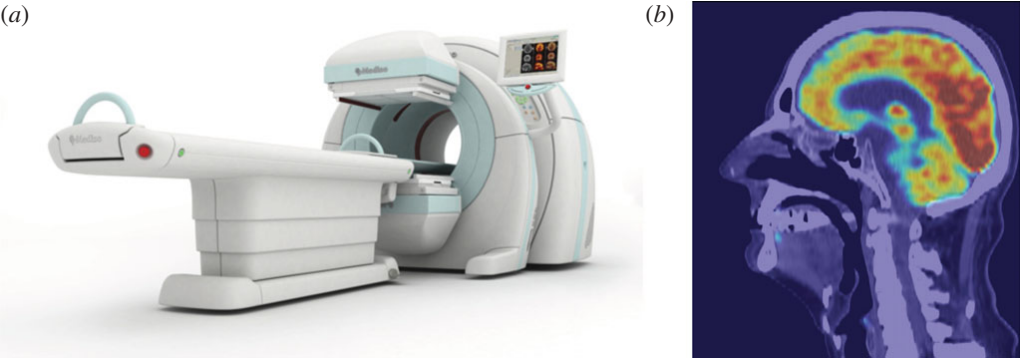
(*a*) PET–CT–SPECT system, courtesy of Mediso. (*b*) Multi-modal PET–MRI image of neck tumour. This medical diagnostic technique uses time-resolved imaging of single photons generated in scintillating crystals when hit by gamma rays that result from nuclear decay.

Biomedical imaging has also profited from time-resolved single-photon sensors. An important example is positron emission tomography (PET) and single-photon emission computed tomography (SPECT), where non-solid-state sensors, such as photomultiplier tubes, are gradually being replaced by silicon photomultipliers (SiPMs), their solid-state counterparts [10]. This process has been accelerated by the introduction of TOF PETs that have much more stringent requirements on the timing accuracy of gamma event detection. Intensive research activity is currently focused on timing resolution, whereas digital SiPMs are approaching the 100 ps limit [11,12]. Note that SiPM-like sensors are also being considered in TOF imagers operating in TCSPC mode, whereas coincidence is used to improve noise and background robustness [13]. Figure 4 shows an example of a neck tumour reconstructed using a commercial PET system. Figure 4 also shows a multi-mode imaging system used to perform PET, computed tomography (CT) and SPECT imaging in rapid succession.

Finally, time-resolved single-photon imaging is also conquering space, thanks to the introduction of vision-assisted docking and time-resolved Raman spectroscopy [14]. Through gating, time-resolved imaging enables one to separate Raman contributions from background and fluorescence responses that are generally several orders of magnitude higher in photon counts [15]. Free-space visible communication may also represent an avenue of research and development for single-photon detection, as shown recently in [16].

## 2. Single-photon detection

### (a) Single-photon detection via avalanching: the single-photon avalanche diode

A class of APDs operating above breakdown, in so-called Geiger mode and known as single-photon avalanche diodes (SPADs) or Geiger-mode APDs, is of particular interest owing to their amenability to integration in planar silicon processes in combination with conventional digital and analogue circuitries.

Although SPAD technology has grown at a fast pace, it is only with the introduction of SPADs fabricated in planar technology in the 1980s [17,18] that it has become possible to miniaturize them, to a certain extent. Several researchers have studied SPADs from an experimental and modelling perspective for at least three decades [19], though no massive arrays were built until the early 2000s. The technology that made this possible was introduced in 2003 [20], with the creation of a fully integrated CMOS SPAD [21]. Massively parallel arrays followed soon after, with the first 8×4 [4] and 32×32 SPAD image sensors [5,22] as well as linear sensors [6].

The next important step towards miniaturization was the migration to submicrometre CMOS technologies [23,24] and later deep-submicrometre CMOS technologies [25–29]. Miniaturization is important for two reasons. First, it enables larger formats; second, it helps improve certain performance measures connected to the number of charges involved in an avalanche, such as crosstalk, afterpulsing and dead time. A discussion on these measures will follow later. For these reasons, the push towards smaller feature size has continued over the past few years with the introduction of SPADs integrated in 90 [30,31] and 65 nm [32,33] CMOS technologies.

CMOS SPADs are not the only solid-state single-photon detector technology; emerging new technologies based on cryogenic nanowires [34], for example, are becoming more and more practical, especially thanks to portable, low-power refrigerating units. However, the number of pixels that can be effectively integrated and read out in this technology is still low, thus more engineering development will be required to achieve the levels of integration guaranteed by CMOS SPADs today. SPADs integrated in new materials, e.g. germanium-on-silicon and InGaAs and InP, are progressively becoming mainstream, where inherent CMOS compatibility [35] and low-temperature post-processing [36] are core techniques. New substrates are also appearing, such as sapphire and germanium, and plastic is being investigated [37]; these trends will pave the way for using single-photon detection in new applications, such as disposable assays, eatable probes and implantable sensors.

### (b) Single-photon avalanche diode basics

**Figure 5.**
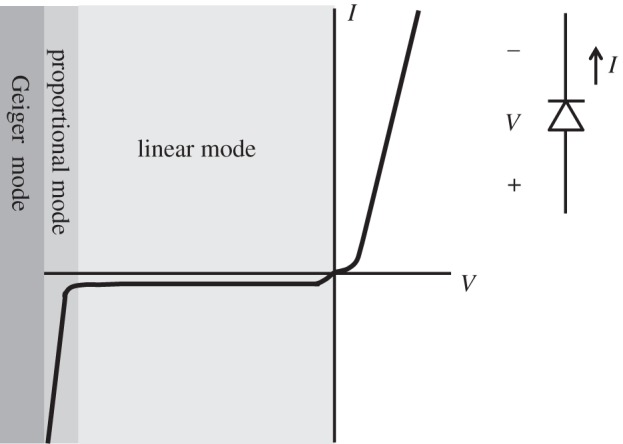
*I*–*V* characteristics of a diode. Conventional photodiodes operate in linear mode, far below breakdown. APDs and SPADs operate, respectively, slightly below and above breakdown, where the optical gain ranges from a few tens of units to infinity.

A CMOS SPAD is essentially a *pn* junction biased above breakdown, in so-called Geiger mode, and equipped with avalanche quenching and recharge mechanisms. Upon photon detection, an avalanche may be triggered. There exist five phases in an avalanche: *seeding*, *build-up*, *spreading*, *quenching* and *recharge*, which concludes the process [19,38]. The seeding occurs when an electron–hole is generated. At this point, the triggering of an avalanche is a non-deterministic process, but a condition must occur by which the mean ionization per free carrier 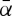 integrated over the depletion region DR exceeds unity; this is known as breakdown integral:

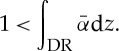
In silicon, the ionization rate is different for electrons and holes, thus the minority carriers in the depletion region determine a different set of properties for the avalanche that develops in it. The voltage at which the breakdown integral reaches unity is known as breakdown voltage, *V*_BD_. In Geiger mode, the *pn* junction is biased above the breakdown voltage by a voltage known as excess bias, *V*_E_. This voltage, if increased, is responsible for a higher electric field across the junction, thus increasing the ionization rates and consequently the probability of an avalanche to trigger. Figure 5 shows the *I*–*V* characteristics of a diode and the various bias conditions used by conventional photodiodes, APDs and SPADs.

Upon the triggering of the avalanche, a build-up phase occurs. During this phase, two processes emerge: a positive feedback from ionization and a negative feedback from drift and coupled resistances, generally dominated by space-charge resistance. The positive feedback is responsible for a rapid growth in local current density, until the current flow across coupled resistances causes the local potential to decrease to the breakdown voltage. This process is internal to the junction, and it is much faster than any voltage changes observable externally. Once the positive and negative feedback processes are balanced locally, the avalanche spreads (spreading phase) via a multiplication-assisted diffusion process towards the extremities of the diode at a speed of about 10–20 μm ns^−1^. Only at this point can the avalanche process be seen externally. Quenching may stop the avalanche now, thus preventing the destruction of the device by overheating.

Quenching can be implemented as an active or as passive process. In active mode, fast circuitries (as simple as a single transistor) are used to bring the bias of the cathode or the anode quickly to a situation in which an avalanche cannot be sustained. After quenching, the same or a different circuitry is used to bring the *pn* junction back to the initial state of above-breakdown biasing, thus enabling it for the next detection. In passive mode, the avalanche current itself is forcing the *pn* junction to return to a bias where the avalanche cannot be sustained; this is achieved by letting the avalanche current flow through a ballast resistance that forces the proper bias. Recharge is achieved by simply recharging the *pn* junction above breakdown through the same resistance until the initial bias is achieved again. The detection/recharge cycle takes a time known as dead time, *t*_dead_, during which the sensor, to a first approximation, is not active. In reality, while the dead time is well controlled in active recharge, the same cannot be said of passive recharge, where the sensor becomes active almost immediately after quenching and its sensitivity grows to reach nominal value when reaching *V*_op_. A review of active and passive quenching/recharge mechanisms can be found in [39].

**Figure 6.**
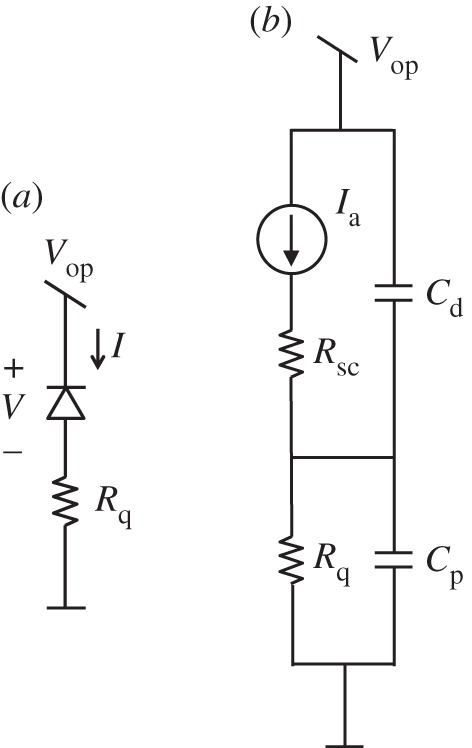
(*a*) SPAD with passive quenching and recharge circuit and (*b*) simple quantitative model. The model includes the main internal parasitic components of a SPAD.

A simple quantitative model for the SPAD is shown in figure 6. In this model, a current source *I*_a_ represents the avalanche current generation process, *R*_sc_ the space-charge resistance seen in an abrupt single-sided *pn* junction and *C*_d_ the parasitic component owing to the depletion region at the junction. *R*_q_ is the quenching resistance used in a passive quenching scheme, and *C*_p_ is the parasitic capacitance at the exterior of the SPAD.

In the model of figure 6, the current source is non-trivially modelled from the free carriers in the diode; owing to the high electric field, these carriers travel at saturation velocity in the junction, rapidly accelerating to that speed upon generation. The current densities obey standard continuity equations and, as the current flowing though the diode increases, the voltage across the current source exponentially drops to *V*_op_−*V*_E_=|*V*_BD_|, thus causing nearly all the current produced by *I*_a_ to flow through *C*_d_ via *R*_sc_. This process models the build-up and it may last up to a few picoseconds; at the end of it, the voltage across the current source stays constant to *V*_BD_ causing *I*_a_ to behave as a voltage source and forcing the voltage at the cathode to follow an exponential behaviour with time decay constant *R*_sc_(*C*_d_+*C*_p_), assuming *R*_sc_≪*R*_q_. This process models the spread. In this model, in principle, the avalanche would continue forever; however, owing to the parasitic inductance to ground (not shown in the model), the current at the anode falls to below a certain level when the avalanche eventually quenches.

**Figure 7.**
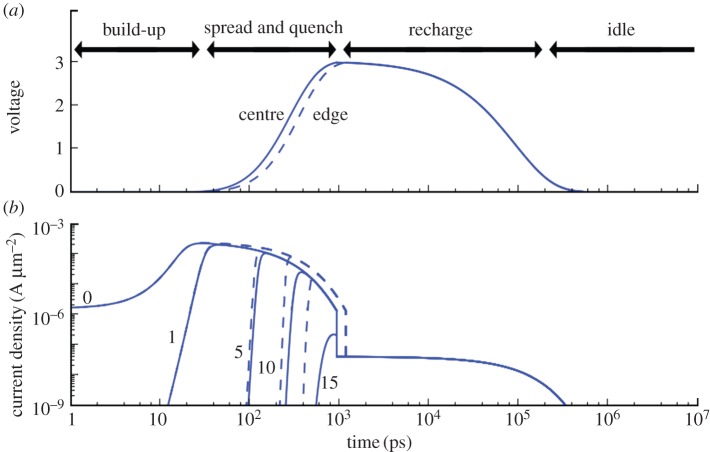
The five phases in the avalanching process: seeding, build-up, spread, quenching and recharge [38]. Voltage at the anode of the *pn* junction as a function of time (*a*); current density as a function of the distance from the junction in steps of 1 μm (*b*). Note the logarithmic time scale. (Online version in colour.)

Upon avalanche quenching, the diode becomes an open circuit and thus the voltage across *C*_p_ must discharge to zero through *R*_q_. This is a classic exponential behaviour with time decays constant *R*_q_*C*_p_; this process models the recharge and it may last a few nanoseconds. This model was extended by Fishburn [38] to characterize larger devices. In this case, while the models for seeding and build-up remain unchanged, avalanche propagation becomes dominated by ionization-assisted diffusion. Figure 7 shows the five phases of the avalanching process in a SPAD as modelled in [38].

### (c) Single-photon avalanche diode implementation in a planar process

**Figure 8.**
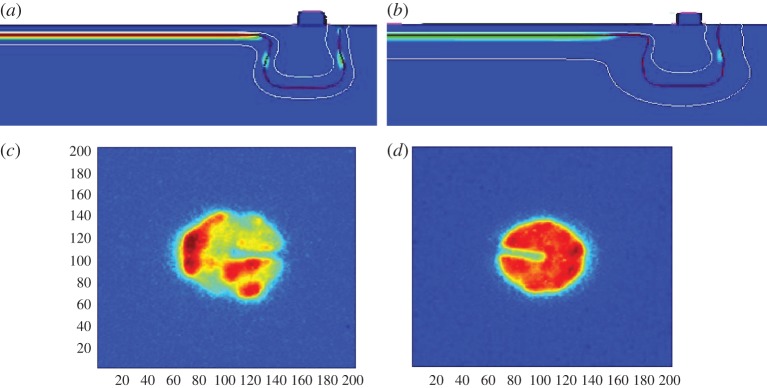
Cross section of planar *pn* junction with electric field simulation (*a*,*b*), where the electric field (arb. units) is plotted near the guard ring. In (*a*), the field exceeds critical values at the edge resulting in PEB, whereas, in (*b*), it does not [27]. The arb. units scale goes from blue (low field) to red (high field). Light emission test: PEB-prone SPAD (*c*); PEB-free SPAD (*d*). The horizontal bar present in both figures is due to the metal connection to the *p*^+^ layer. The arb. units scale goes from blue (low emission) to red (high emission). (Online version in colour.)

A conventional *pn* junction is implemented in a planar process through implantation and annealing. When applying a high voltage in reverse bias, the electric field at the junction is maximized at the corners of the junction (figure 8). This has the effect of a preferential avalanching probability in these locations, thus causing premature edge breakdown (PEB). As a result, a SPAD becomes sensitive only in a small section of its surface. Figure 8 shows two SPAD implementations, where the guard ring was ineffective (figure 8*a*,*c*) and effective (figure 8*b*,*d*) to prevent PEB.

A technique called light emission test (LET) enables one to visualize the location of avalanches occurring in a period of time. The technique uses the fact that impact ionization generates photons, with a certain probability, and thus avalanche discharges can be optically identified. Figure 8 shows an LET on two different devices in which PEB suppression was, respectively, unsuccessful and successful. Several techniques exist to implement PEB prevention. The common denominator is the reduction of the electric field at the edges and everywhere else in the device, so as to maximize the probability that the avalanche is triggered in the centre of the multiplication region. This is the region where the critical electric field for impact ionization is reached and, possibly, exceeded; in silicon, this field is approximately 3×10^5^ V cm^−1^.

**Figure 9.**
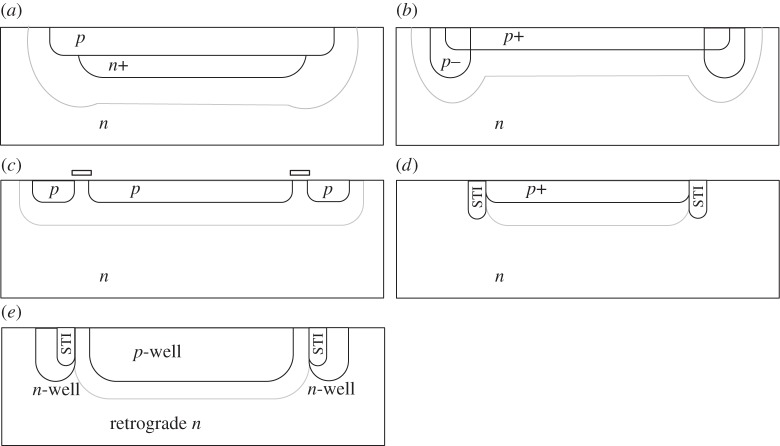
Premature edge breakdown prevention mechanisms in planar and semi-planar processes. (*a*) Mechanism was first proposed by Spinelli *et al.* in [40] and (*b*) by Cova *et al.* in [17]. (*c*) Mechanism was first proposed theoretically by Pauchard *et al.* in [41] and implemented by Niclass *et al.* in [25] and by Fishburn in [38], whereas (*d*) was first proposed by Finkelstein *et al.* in [26]. Gersbach *et al*. [27] proposed to encapsulate the STI in multi-layered doped semiconductor material in order to force trap-generated carriers to recombine before reaching the multiplication region. (*e*) Mechanism was proposed by Richardson *et al.* in [28] and by Webster in [42]. The grey line represents the limit of the depletion region, within which multiplications can occur.

In figure 9, five of the most used structures are shown; the structures show the edge of the *pn* junction, where it is assumed the *pn* junction to be round-shaped even though other shapes are also used, such as squares, rounded rectangles, hexagons and octagons. In figure 9*a*, the *n*^+^ layer maximizes the electric field in the middle of the diode [40]. In figure 9*b*, the lightly doped *p*^−^ implant reduces the electric field at the edge of the *p*^+^ implant; this structure is commonly known as *guard ring* [17]. In figure 9*c*, a floating *p* implant locally increases the breakdown voltage. With a polysilicon gate one can further extend the depletion region (grey line in figure 9) [25,38,41]. This design may also be used to create implicit guard rings by enclosing substrate regions with wells. Enclosure is achieved by placing wells so close to each other that they merge at the bottom, thus creating a substrate enclave. In a process with shallow or deep trench isolation (STI, DTI), it is possible to decrease the electric field using the geometry of solution (figure 9*d*); this solution, however, suffers from high noise owing to trapping centres induced by trench fabrication [26]. Thus, one needs to adopt techniques to prevent traps accumulated in the trench during fabrication from inducing PEB. An effective technique proposed in [27] consists of using several layers of doped semiconductor material with decreasing doping levels from the trench to the multiplication region. The purpose is to achieve short mean free paths close to the trench, thereby forcing carriers generated there to recombine before reaching the multiplication region. In figure 9*e*, a deep *p*-well is used to establish a deep junction, below which the multiplication takes place, and a retrograde deep *n*-well establishes a lightly doped region at the surface to act as an implicitly defined lightly doped guard ring [28,42].

**Figure 10.**
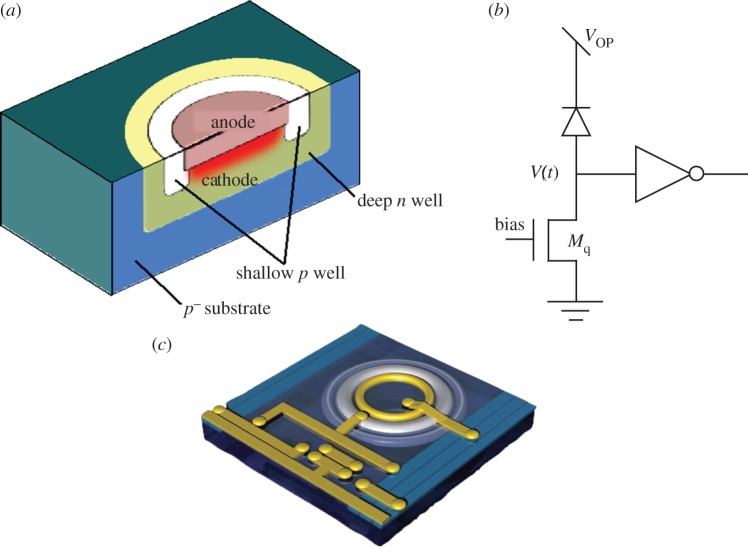
(*a*) SPAD cross section in a conventional CMOS process with the multiplication region highlighted. (*b*) Passive quench and recharge circuitries as well as pulse shaping. (*c*) Artist’s rendering of complete SPAD layout.

In the remainder of the paper, we focus our attention on the schemes in figure 9*a*,*b*,*e*, because they require, in general, no modifications to the process and thus enable the design of large SPAD array chips in standard CMOS technologies. Figure 10 shows the cross section of a SPAD implemented in a conventional CMOS process. The structure comprises a *p*^+^ implant obtained from a transistor footprint and a type-b guard ring obtained, for example, from a shallow *p*-well all encapsulated in a deep *n*-well for isolation purposes. Figure 10 also shows how to implement the quenching resistor via MOS transistor *M*_q_ biased in the linear region. The cathode (in this case) generates a voltage pulse *V* (*t*) similar to the one shown in figure 7; it can be shaped with a comparator or a simple inverter, as shown in the figure. An artist’s rendering of the layout of the SPAD is shown in the figure.

### (d) Quenching and recharge

When an avalanche has occurred, quenching must be performed as soon as possible, so as to reduce carriers involved in the avalanche. Fewer carriers generate smaller photon fluxes, thus reducing optical crosstalk; fewer carriers mean lower probability of afterpulsing owing to the lesser probability of trap occupation. As mentioned earlier, quenching may be implemented with a ballast resistor (figure 6), whereas active quenching is generally implemented via a mechanism activated upon detection of the avalanche.

Recharge can be performed passively or actively. In passive recharge, dead time is controlled poorly owing to the variability and nonlinearity of the ballast resistance. As a result, a number of active recharge techniques have been investigated. The literature on the subject of active/passive quenching/recharge is extensive and it is beyond the scope of this paper.

**Figure 11.**
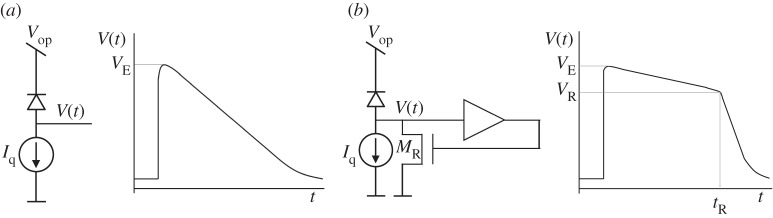
Active recharge mechanisms: (*a*) single-slope and (*b*) double-slope. In single-slope recharge, a current (*I*_q_) controls the rate of the recharge; the recharge is completed in *CV*_E_/*I*_q_. In double-slope recharge, a small threshold is used to quench the SPAD; it subsequently recharges through *I*_q_, until a second threshold is reached, causing a rapid recharge through switch *M*_R_.

In figure 11, the concept of two active recharge mechanisms is reported. In single-slope recharge, dead time is controlled precisely, thus enabling one to avoid, in principle, the overlapping of subsequent pulses, with a consequent underestimation of photon counts. Double-slope active recharge is also used to control dead time; however, in this design, the SPAD is artificially biased below breakdown during the entire recharge time, thus preventing avalanche creation, unlike in single-slope recharge, where partial sensitivity is present during recharge [8,9]. The SPAD’s dead time is effectively controlled by time *t*_R_ at which the second slope is activated. If the voltage *V*_R_ achieved at this point still disables the avalanche, then it is guaranteed that the device is still in the dead time regime.

**Figure 12.**
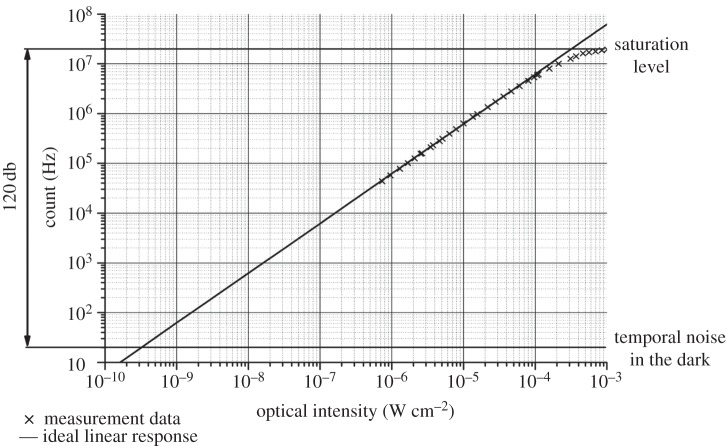
Photo response in actively recharged SPADs [4], where saturation frequency is *f*_sat_=1/*t*_dead_. Passively recharged SPADs reach saturation at *f*_sat_= 1/e⋅*t*_dead_. A higher saturation is reached by active recharged SPADs as the generated pulses do not merge to reduce, as a result, the overall photon counts.

Overlapping avalanche pulses has the effect of lowering the upper limit of the photon flux detectability, *f*_sat_, by 1/e, i.e. *f*_sat_=1/e⋅*t*_dead_. In an actively quenched SPAD on the contrary, *f*_sat_=1/*t*_dead_. Moreover, the photo response of a passively recharged SPAD drops after saturation is achieved due to the avalanche pulses fusing with each other, thus preventing proper counting [8,43]. Figure 12 shows the typical photo response in a digitally counting SPAD array.

## 3. Single-photon avalanche diode-based image sensors

### (a) From single-photon avalanche diode to pixel

**Figure 13.**
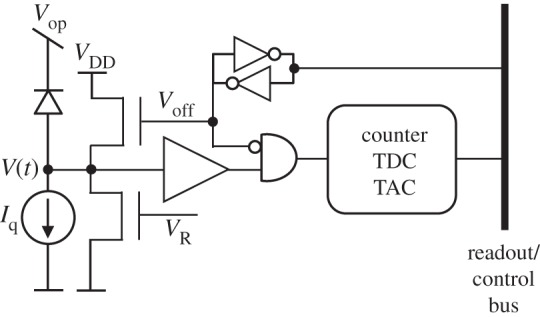
Generic pixel and its components. Screamers are turned off by setting an on-pixel memory via the readout/control bus. Analogue and digital counters can be used for uncorrelated photon counting, whereas correlated photon counting requires a TDC or a TAC.

A SPAD generates a digital pulse upon detection of a photon; the pulse can drive a digital [44] or an analogue [45] counter. The counted values are generally read out sequentially, whereas the analogue counter requires further A/D conversion with a resolution of at least the maximum number of states, or the counting range. A pixel usually comprises a SPAD ensemble (including quenching and recharge), a gating and a masking mechanism, a counter and a readout interface. Figure 13 shows a generic pixel and its individual components. The gating mechanism is used to turn off the SPAD when it is not needed, so as to reduce power consumption, and, most importantly, noise. Figure 13 shows a possible gating circuit based on a pull-up transistor controlled by *V*_off_ and intended to bring the SPAD out of Geiger mode of operation. The SPAD is then re-enabled by way of the recharge transistor controlled by *V*_R_, which is generally a short voltage pulse. Masking is used to turn off those SPADs that have excessive noise levels and it is implemented via a memory that controls *V*_off_ and an AND gate. A counter (digital [46,47] or analogue [48,49]) may be replaced by other components, such as time-to-digital converters (TDCs) or time-to-amplitude converters (TACs). The readout interface is designed to transfer the content of the pixel to the exterior of the chip to a digital or analogue bus by way of serialization and parallelization techniques.

Analogue counters, based on injecting or extracting a well-defined charge packet in a capacitance upon the SPAD firing, have recently gained traction, thanks to the capability of designers to keep the fill factor high even in relatively small pixels, while achieving high counting resolutions [45]. The drawback is the need to implement ADCs to reconvert the analogue signal read out from each pixel onto a digital code; however, the ADC specifications may be relaxed given the nature of the signals being processed and advantageous power–speed trade-offs may be exploited.

### (b) Single-photon avalanche diode pixel performance parameters

Individual SPADs are characterized by their sensitivity, measured as *photon detection probability* (PDP); the noise performance is measured as a rate of spurious pulses owing to thermal events or *dark count rate* (DCR). Other parameters include *timing jitter*, also known somewhat inappropriately as *timing resolution*, which measures the uncertainty of a photon detection in standard deviation from a Gaussian fit or full-width-at-half-maximum (FWHM) of the same, *afterpulsing probability*, and, as mentioned earlier, *dead time*.

**Figure 14.**
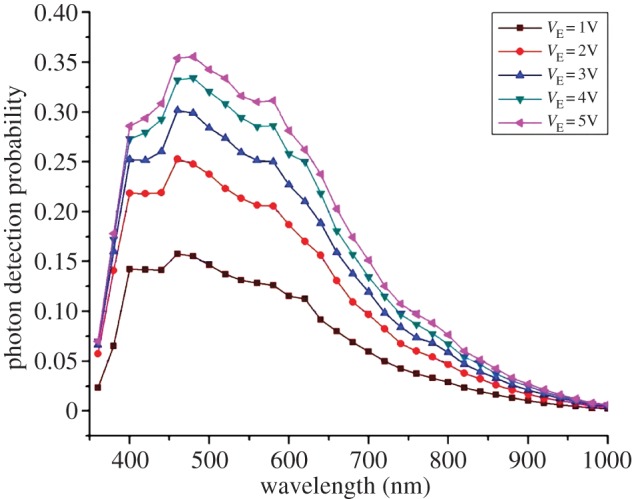
PDP as a function of excess bias and wavelength in a 130 nm SPAD at room temperature [27]. (Online version in colour.)

PDP is a function of excess bias and wavelength; in CMOS SPAD implementations, the sensitivity range is mostly in the visible spectrum, with somewhat reduced near infrared and near ultraviolet response. Figure 14 shows a plot of PDP as a function of excess bias and wavelength in a 130 nm SPAD [27].

**Figure 15.**
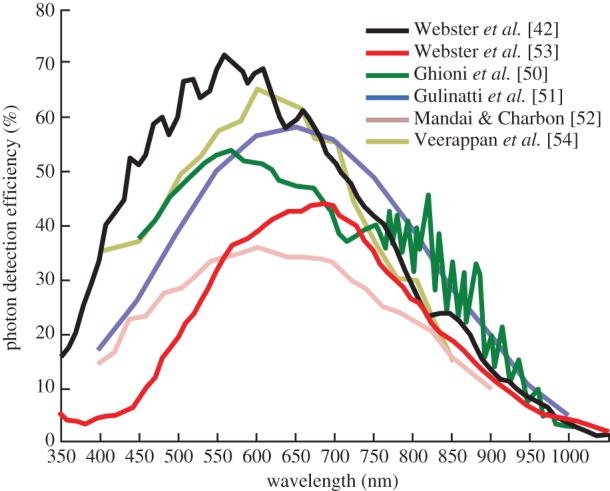
PDE (assuming FF=1) found in the literature for an indicated excess bias voltage [42,50–54]. (Online version in colour.)

Photon detection efficiency (PDE) is often used to characterize sensitivity. The relations between PDE, PDP and quantum efficiency are as follows:

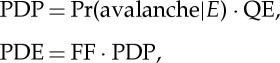
where FF is the ratio between active area and total pixel area, or fill factor, and QE is the quantum efficiency. Pr(avalanche|*E*) is the probability that an absorbed photocarrier (an event *E*) originates an avalanche [38]. Figure 15 shows a comparison between PDE values found in the literature at room temperature and for a given excess bias voltage as published in that literature.

**Figure 16.**
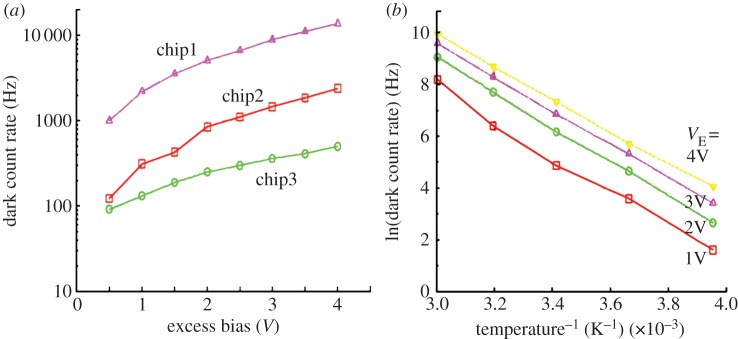
DCR as a function of excess bias in three chips (*a*). DCR as a function of temperature and excess bias voltage *V*_E_ in an Arrhenius plot (*b*). The measurements are derived from [52]. (Online version in colour.)

Dark counts, characterized in terms of the average rate of occurrence or DCR, are due to two main mechanisms, trap-assisted counts and band-to-band tunnelling, or a combination of these two phenomena. DCR is a function of excess bias as well as temperature. At low temperatures, band-to-band tunnelling dominates, whereas at high temperatures, trap-assisted dark counts dominate. The phenomena have been analysed systematically in the literature, and further discussion is beyond the scope of this paper; figure 16 shows the dependency of DCR on temperature and excess bias as measured in [52]. Figure 16 also shows the variability of DCR from chip to chip in this particular CMOS process.

**Figure 17.**
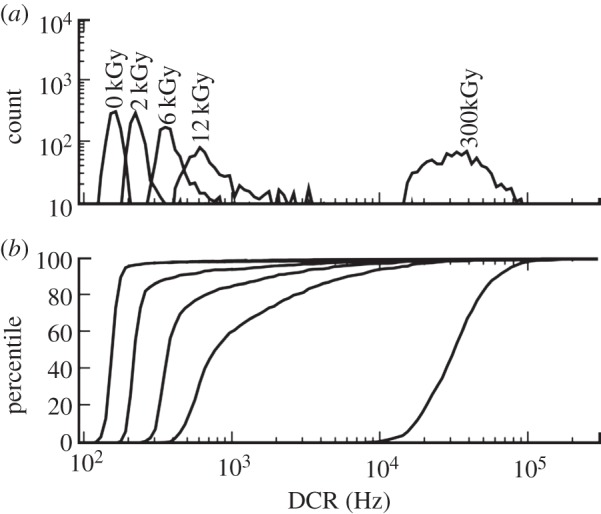
Dependency of DCR distribution upon exposing a SPAD to gamma radiation generated from a Co-64 source [38]. (*a*) The DCR distribution for various doses from 0 to 300 kGy. (*b*) The DCR cumulative distribution in SPADs fabricated in 0.35 μm CMOS technology.

As trapping centres may be increased in number by the exposure of the SPAD to certain ionizing radiation, DCR may also increase and its distribution change as a function of radiation dose. Fishburn conducted such an experiment with proton and gamma radiation exposure. The experiments showed that the median of DCR increased with the dose while the distribution spread also increased, owing to the increase of trapping centres in the substrate (figure 17).

**Figure 18.**
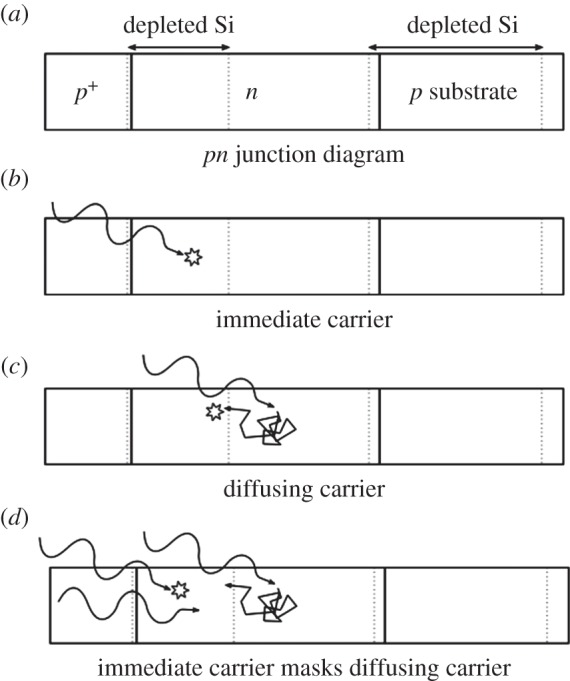
Timing jitter mechanism. (*a*) Structure of a slice of the SPAD. (*b*) Immediate carrier multiplication. (*c*) Diffusing carrier followed by multiplication. (*d*) Combination of multiple processes [38].

**Figure 19.**
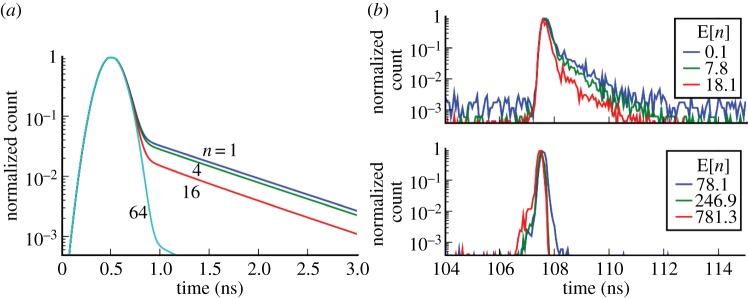
Typical timing jitter response in a SPAD: (*a*) simulated and (*b*) measured response as a function of the number of detected photons *n*. The response is the result of the superimposition of Gaussian statistics and an exponential tail. The latter becomes less relevant with the increase of detected photons; hence, jitter is reduced by higher photon fluxes. In the measurements, the number of detected photons is expressed in terms of their expected value E[*n*], owing to the statistical measurement involved [38]. (Online version in colour.)

Timing jitter is caused by a complex mechanism involving immediate carrier multiplication, multiplication after carrier diffusion and a combination of multiple processes. Figure 18 illustrates the processes and combinations thereof described in detail in [38]. The statistics of these processes are different, and the cumulative result can be roughly described as the superimposition of a Gaussian response and an exponential tail. The relative importance of one process versus the others is related to the number of detected photons that statistically contribute, depending on the depth at which they are detected, to one or the other. In general though, higher fluxes result in reduced jitter as shown in the simulated and measured results in figure 19, derived from the work of Fishburn [38].

**Figure 20.**
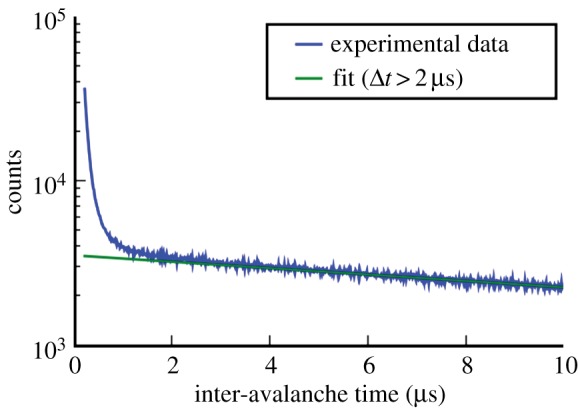
Afterpulsing characterized as a histogram of interarrival times Δ*t* in a typical SPAD after [38]. Afterpulsing relates to the presence of secondary avalanches triggered by the primary ones by trapping and other device non-idealities. (Online version in colour.)

Afterpulsing is a process by which a primary avalanche is followed by other avalanches unrelated to photons owing to traps in the device lattice and other non-idealities. Afterpulsing characterization is performed by measuring interarrival times in a SPAD at given excess bias, temperature and illumination levels. Figure 20 shows the histogram of interarrival times measured in a SPAD; the area enclosed between super-exponential response and extrapolated exponential response (straight line in the figure) is proportional to afterpulsing probability as discussed in detail in [38]. The physical process underlying afterpulsing has been thoroughly researched in the literature. Afterpulsing’s typical cause is also related to trapping centres that cause carriers to be released at a random time after an avalanche, thus causing a spurious avalanche; more details can be found in [38].

The parameters discussed above have appeared in the literature for individual SPADs implemented in a variety of CMOS processes [22–62]. Some performance indicators found in individual SPADs are described in table 1 for six different SPAD implementations in CMOS submicrometre and deep-submicrometre processes. SPADs have been often characterized based on a number of figures-of-merit that capture their overall performance. A good example of such an approach can be found in [63].

**Table 1.** Comparison of SPAD performance for a selection of front side-illuminated CMOS and CMOS image sensor (CIS) processes. All the measurements refer to room temperature, unless otherwise indicated.

	measurement	0.8 μm [22]	0.35 μm [23]	180 nm [52]	130 nm [27]	130 nm [28]	130 nm [42]	unit
	jitter (FWHM, *λ*: 637 nm)	82^a^	80	165 (*λ*: 790 nm)	125	200 (*λ*: 815 nm)	60 (*λ*: 654 nm)	ps
	DCR (mean at 300 K)	350 (*V*_E_: 5.0 V)	750 (*V*_E_: 3.3 V)	2500 (*V*_E_: 4 V)	220 (*V*_E_: 2.0 V)	25 (*V*_E_: 1.4 V)	4000 (*V*_E_: 12 V)	Hz
	active area	38	78	78.5	58	50	50	μm^2^
	mean DCR p. active area	9.2	9.6	31.8	3.8	0.5	80	Hz μm^−2^
	breakdown (*V*_BD_)	25.5	17.4	19.7	12.8	14.4	20	V
	dead time	<40	40	—	100	100	—	ns
	PDP at peak	26 (*V*_E_: 5.0 V)	40 (*V*_E_: 3.3 V)	36 (*V*_E_: 4 V)	36 (*V*_E_: 2.0 V)	28 (*V*_E_: 1.4 V)	72 (*V*_E_: 12 V)	%
	peak *λ*	480	450	600	480	500	560	nm
	technology	CMOS	CMOS	CMOS	CIS	CIS	CIS	—

^a^This result was reported after the publication of [22] as an improvement of the published value.

### (c) Characterization of arrays of single-photon avalanche diodes and single-photon avalanche diode image sensors

**Figure 21.**
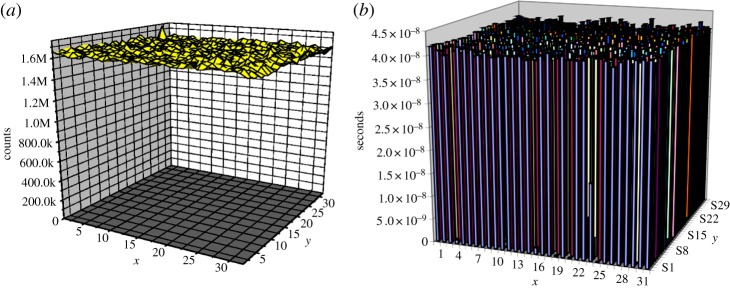
(*a*) PDP and (*b*) dead time uniformity in a 32×32 array of low-pitch passively recharged pixels. PDP variations in the sensor are due to localized breakdown voltage variations, whereas dead time non-uniformity is due to localized variations of parasitics in the recharge circuit of each SPAD. (Online version in colour.)

When implemented in an array, other performance measures become relevant to the quality of the imager. Because of the importance of dead time to *f*_sat_, for example, dead time uniformity is crucial to a good quality sensor. PDP uniformity is also important along with timing jitter uniformity in applications where lifetime is used as a discrimination factor such as in FLIM. Crosstalk and afterpulsing have to be accounted for at the sensor level and properly characterized at various temperatures and excess bias voltages. Figure 21 shows the dead time and PDP uniformity achieved in a 32×32 pixel array at a given temperature and excess bias [22]. The relation between PDP and these two parameters is complex, and the literature is quite thorough in this subject; see [38] for a review.

**Figure 22.**
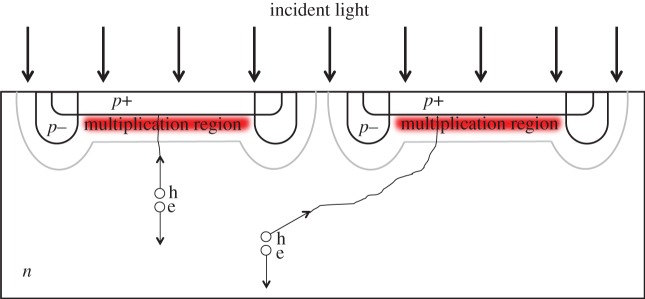
Electrical crosstalk mechanism owing to substrate photocarrier exchange. Upon photon absorption, the electron–hole pair is accelerated opposite to each other. The minority carrier is drifting to the depletion region until multiplication can occur; this process, however, may take place in an adjacent pixel thus creating crosstalk. The figure illustrates two photocarriers one of which creates crosstalk. (Online version in colour.)

Crosstalk may be electrical and/or optical. Electrical crosstalk is due to the electrical interference between pixels. It may be caused by a temporary drop of sensitivity and DCR in a victim pixel owing to the drop of excess bias voltage. The latter, in turn, may be caused by a neighbouring aggressor pixel as an avalanche is triggered. Similarly, substrate noise carriers or photocarriers originated in one or more pixels may be picked up by the victim pixel and a spurious avalanche may thus be triggered, as shown in figure 22.

**Figure 23.**
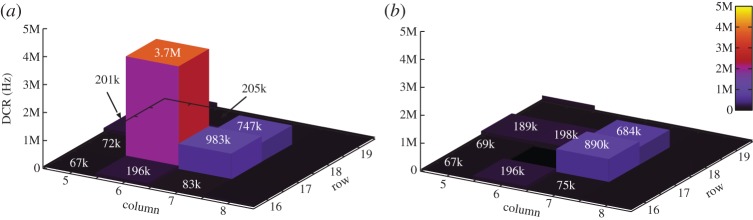
Crosstalk characterization around a high DCR pixel before (*a*) and after suppression of that pixel (*b*) [12]. Crosstalk was measured as variation of count rate before and after the suppression of a SPAD, generally a high-noise SPAD or screamer. Alternatively, cross-interarrival analysis in pairs of SPADs can also be used to obtain the same result. (Online version in colour.)

Optical crosstalk may occur when an avalanche is triggered in the aggressor pixel. By impact ionization, several photons may be emitted, thus causing a victim pixel to detect them. While electrical crosstalk is strongly dependent on the design of supply lines and of substrate noise rejection measures, such as decoupling capacitances and resistive/inductive supply buses, optical crosstalk may only be influenced by the number of carriers involved in an avalanche and by pixel pitch. The reduction of the number of avalanching carriers may be best achieved by reducing the active area of a SPAD, and thus its capacitance at a cost of lower fill factor if the pixel pitch is kept constant [22]. Figure 23 shows the crosstalk effects on neighbours to a high activity pixel before and after suppressing it. Crosstalk in this experiment was measured in terms of counts in addition to the background owing to dark counts and basic uniform illumination. Crosstalk may also be measured in terms of interarrival times between pulses in crosstalking pixels [38], whereas a behaviour similar to afterpulsing with zero dead time is observed.

**Figure 24.**
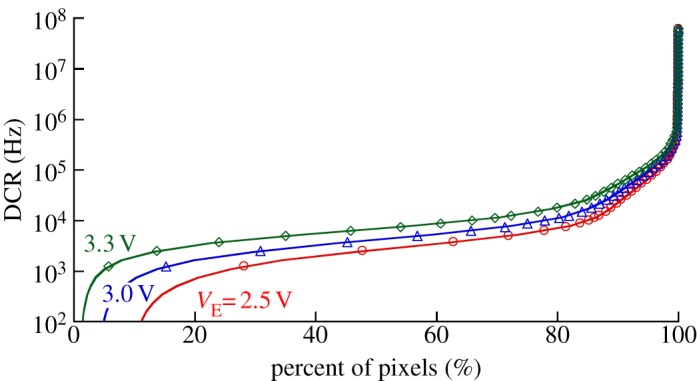
DCR cumulative distribution in a 0.35 μm CMOS process as a function of excess bias [14]. The distribution shows a two-knee behaviour typically observed in most SPAD technologies. By suppressing all those SPADs to the right of the first knee, generally about 15–20% of the SPAD population, a significant improvement of the noise performance of an array can be achieved. Note that the knees in the DCR distribution are generally independent of excess bias voltages. The second knee represents the boundary to screamer pixels that generally represents 0.5–1% of the entire pixel population. (Online version in colour.)

When analysed as an ensemble, pixels may exhibit different noise performance and thus DCR must be analysed in a statistically relevant fashion. Figure 24 shows a plot of the cumulative distribution of DCR measured in a large population of pixels. The 50% line corresponds to the median DCR, whereas on the right, a small population of noisy pixels, known as ‘screamers’, is shown. Although not contributing directly to the median, screamers are often sources of disruption in the SPAD array, causing increased DCR by crosstalk and potentially readout disruptions; they are thus generally removed by masking techniques (figure 13).

**Figure 25.**
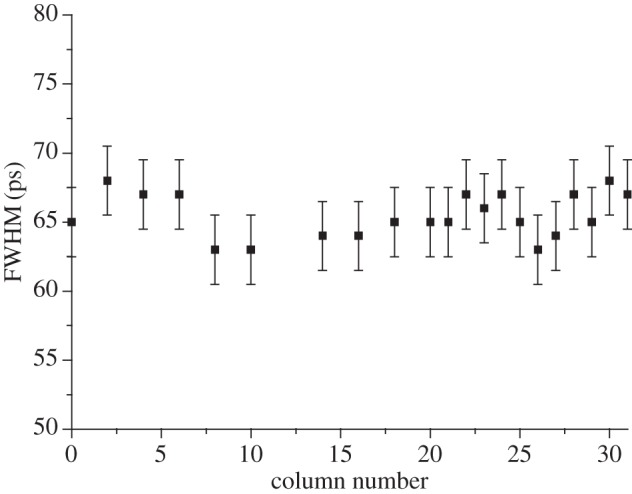
Timing jitter performance uniformity: FWHM timing jitter over an array of 32 SPADs. A detailed discussion of the avalanching models and the resulting time response can be found in [19] and [38].

As mentioned earlier, SPAD image sensors may be effectively used in applications where the photon arrival time must be determined precisely. Thus, timing jitter is an important parameter. Figure 25 shows the timing jitter non-uniformity on an array of 32 SPADs implemented in 0.8 μm CMOS technology. Figure 25 shows a non-uniformity of less than ±5 ps (peak-to-peak) over the whole array integrated on the same chip and measured by exposing the chip to a cone of light from a pulsed laser source. In this case, a femtosecond Ti : sapphire laser source doubled to achieve a wavelength of 488 nm was used.

## 4. Single-photon avalanche diode image sensor architectures

### (a) Architecture versus application

Unlike conventional diodes, SPADs cannot hold a charge proportional to the overall photon count, but they generate pulses in correspondence to photon arrivals; they thus must be handled *in situ*. An example is photon time of arrival (TOA); it too must be performed upon photon detection, requiring advanced architectures that are capable of implementing parallelism or resource sharing.

Possible architectures are (i) in-pixel, (ii) in-column and (iii) on-chip counting. When in-pixel architectures are used, all the operations are performed and saved locally; the stored value is read out later in random access or sequential mode. In-column or cluster counting implies the sharing of operations of all the pixels on the column or the cluster, whereas the result is stored in a column-based memory and read out on a column-by-column basis. When sharing is used, trade-offs between pixel utilization, column/cluster size and detection bandwidth are generally to be foreseen. In these cases, understanding application specifications is key to an appropriate use of the available techniques. On-chip counting or TOA is essentially an extension of the in-column architecture, whereas the working cluster is the entire chip. Similar trade-offs are also used in this case.

### (d) Random access readout

**Figure 26.**
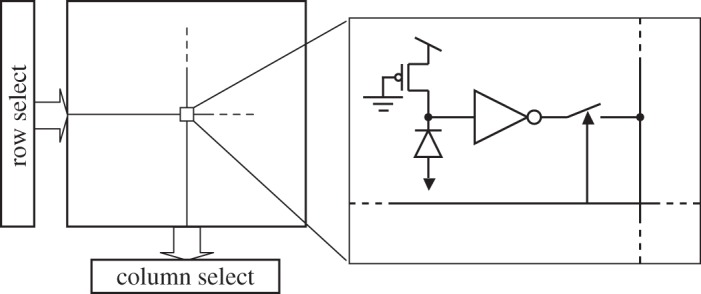
Block diagram and pixel schematic of the 32×32 SPAD array with random access readout.

The first option is to read a pixel at a time, thus ignoring the other pixels. A design demonstrating this feature, and the first implementing large SPAD arrays, comprised a matrix of 32×32 pixels, each with an independent SPAD, a quenching mechanism, a pulse shaping and column access circuitry [5,22]. Owing to the use of random access readout, all time-sensitive operations had to be performed off-chip and an overall jitter as low as 70 ps was measured on a pixel while the entire array was operating. In this design, only one pixel can be read out at any time while photons whose wave function collapses outside that pixel are lost. The simplified block diagram of the imager and the pixel schematic is shown in figure 26.

**Figure 27.**
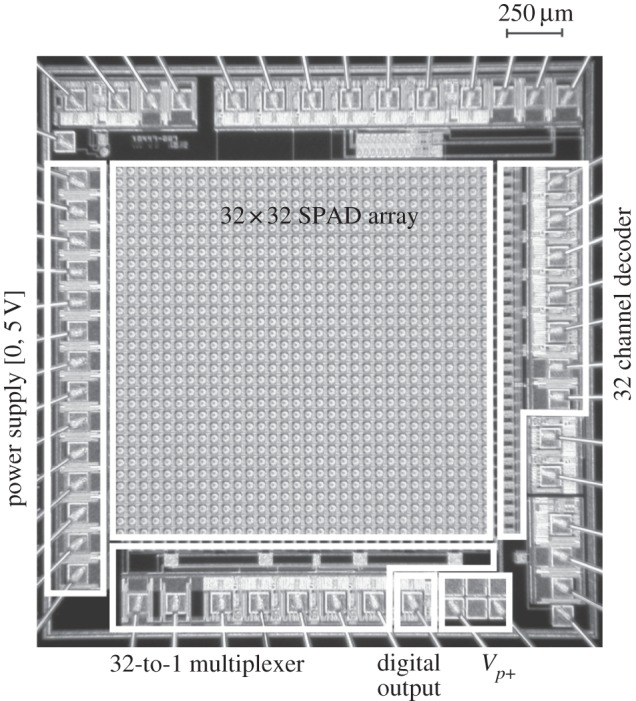
A 32×32 SPAD array with random access readout [5,22]. The chip was implemented in 0.8 μm CMOS technology.

Note that the SPAD anode is connected to a negative voltage and quenching is performed at the cathode via a PMOS. The negative voltage is chosen so as to ensure that the device operates above breakdown by an excess voltage *V*_E_. Thus, the avalanche pulse must be inverted, in this design, with a simple logic inverter with an appropriate threshold. The micrograph of the chip is shown in figure 27.

### (c) Event-driven and latchless pipeline readout

Several techniques have been devised to alleviate the bottleneck of random access readout schemes; we present two of the most successful approaches. The first, known as *event-driven readout*, uses the column as a bus, addressed every time a photon is detected. The address of the row where the photon was detected is propagated to the bottom of the column where a TDC or a TAC may be used, either off chip [23,24,64] or on chip [65]. The second approach, known as *latchless pipelined readout*, consists of using the column as a timing-preserving delay line [66]. If impinging in a certain ‘gate of time’, every photon may trigger a pulse that is injected onto the pipeline at a precise location that corresponds to the physical place where the pixel is located. The row information is thus encoded in the timing of the pulse arrival at the end of the pipeline, thus it can be sequentially reconstructed by a single TDC, which is a sort of miniaturized high-speed chronometer, at the bottom of the column. The TDC will also detect the exact TOA of the photon within a predefined window of time.

**Figure 28.**
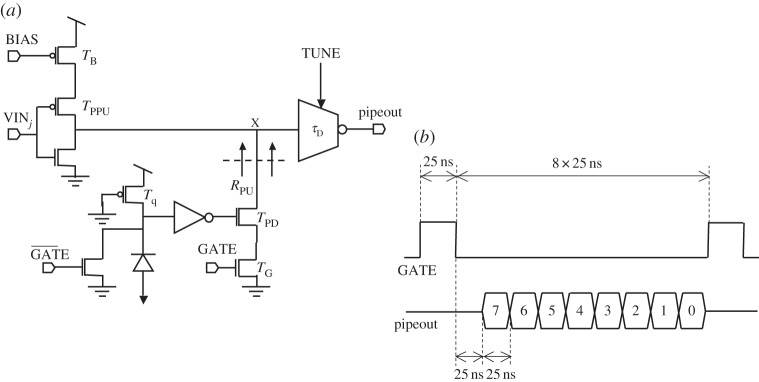
Schematic diagram of the latchless pipelined readout (*a*); timing diagram and operation of the circuit (*b*). The detailed description of the pipeline operation, including the symbols used in the schematic and the signals seen in the timing diagram, are described in the corresponding text.

Figure 28 shows a schematic diagram of the pixel and of the latchless pipeline readout [66]. The avalanche current produced by the SPAD is sensed and converted onto a digital voltage pulse by an inverter. The L to H transition at the inverter’s output pulls down node ‘X’ through transistor *T*_PD_ and resistor *R*_PU_, provided that gating transistor *T*_G_ is enabled by signal ‘GATE’. The anode of the diode is intentionally set to a negative voltage, as discussed above. *T*_q_ was sized for a dead time *τ*_dead_ of 40 ns and by choosing a gating window *τ*_G_ that satisfies inequality *τ*_G_<*τ*_D_<*τ*_dead_. When there is no activity on the preceding delay line, signal ‘VIN_*j*_’ is at logic level L, hence the gate of source-degenerated transistor *T*_PPU_ is L, thus the impedance at node ‘X’ is dominated by the impedance at the drain of *T*_PPU_. When a photon is detected, a pulse is originated at this point and it is propagated towards the remainder of the delay line. When there is activity on the delay line, a logic transition L to H on ‘VIN_*j*_’ occurs, thus causing ‘X’ to become a low impedance node. During this time, any photon detection in this stage will have no effect on travelling pulses, but it will inject spurious pulses onto the line when it is at logic level L, hence the need for gated SPAD operation. To avoid ghost pulses, an appropriately sized NMOS was added to the cathode of the diode. A simplified timing diagram to operate the eight-stage delay line is shown in the figure. Controls ‘BIAS’ (transistor *T*_B_) and ‘TUNE’ are used for coarse- and fine-tuning of the delay line, respectively. The goal is to compensate for technological variations and temperature.

**Figure 29.**
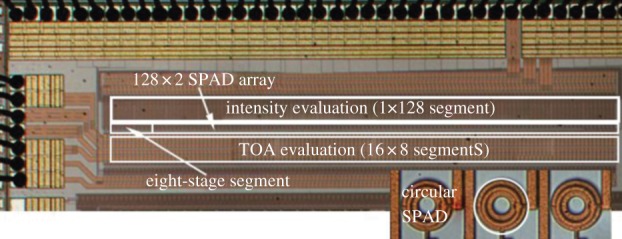
Demonstrator of latchless pipelined readout implemented in 0.35 μm CMOS technology [66] with SPAD pixels in inset. The chip consists of an array of 16×8 segments of SPADs with an independent readout capability per segment. (Online version in colour.)

A chip implementing this concept in a 128×2 SPAD array is shown in figure 29; the architecture was implemented in 0.35 μm CMOS [66]. The chip also includes a single SPAD line for a simple eight-bit time-uncorrelated photon-counting (TUPC) mode.

An alternative technique for event-driven processing of signals produced by SPADs in an on-demand fashion was proposed in [67]. The technique is based on a phase-domain sigma delta approach, similar to an oversampled A/D converter loop, where part of the loop is in the pixel, and the decimation is implemented at column level, thus enabling highly efficient organization of the real estate for detection and processing of TOF information.

### (d) Parallel processed single-photon avalanche diode image sensors

#### (i) LASP

**Figure 30.**
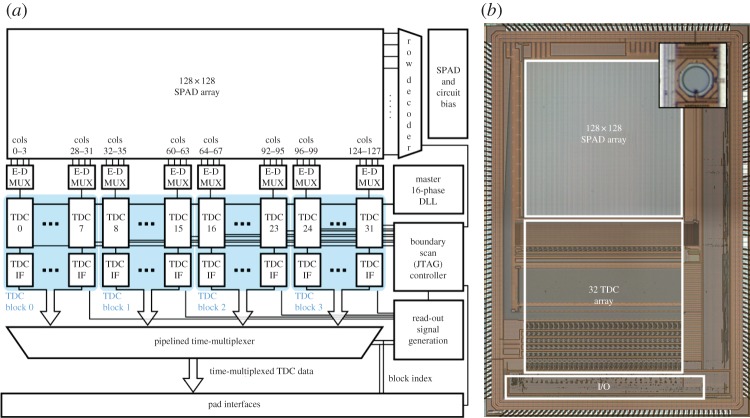
LASP block diagram; it is a fully integrated SPAD array with a bank of TDCs (*a*); photomicrograph of the chip implemented in 0.35 μm CMOS technology (*b*). The inset shows the pixel [65]. The chip has a bank of 32 independent TDCs each of which is responsible for time-of-arrival detection in four columns. A high-speed readout circuit transfers all computed time of arrivals to the outside of the chip at 3.2 Gb s^−1^. (Online version in colour.)

The first design implementing parallel on-chip time discrimination was LASP [65], a 128×128 SPAD array with a bank of 32 TDCs operating simultaneously. Figure 30 shows the block diagram of LASP; a row of 128 SPADs can be randomly selected for TOA processing using a row encoder. A bank of 32 TDCs shared on a four-to-one basis is used for the time conversion to digital code. Each TDC can generate 10 million samples per second (MS s^−1^) with a time resolution of 97 ps.

The TDC implemented in LASP operates in cascade mode, generating the two MSBs via a clocked counter, four intermediate bits with a phase interpolator controlled by a temperature-compensated DLL, and four LSBs by means of a Vernier line of 32 elements to counter metastability. The total time resolution of 10 bits is subsequently routed to the exterior of the chip through a high-speed digital network operating at 3.2 Gb s^−1^. The differential and integral nonlinearity (DNL, INL) of the TDCs was evaluated in detail in [65] to be in a range of ±0.2 and ±1.2 LSB, respectively. The dead time was fixed to 100 ns to allow a complete time-to-digital conversion at reasonable afterpulsing levels. The uniformity of PDP and its spectral behaviour as well as the chip DCR were consistent with the data reported in [23]. The main drawback of the LASP architecture is the need of row selection, thus making it less efficient as only one row is active at any time.

#### (ii) SwissSPAD

**Figure 31.**
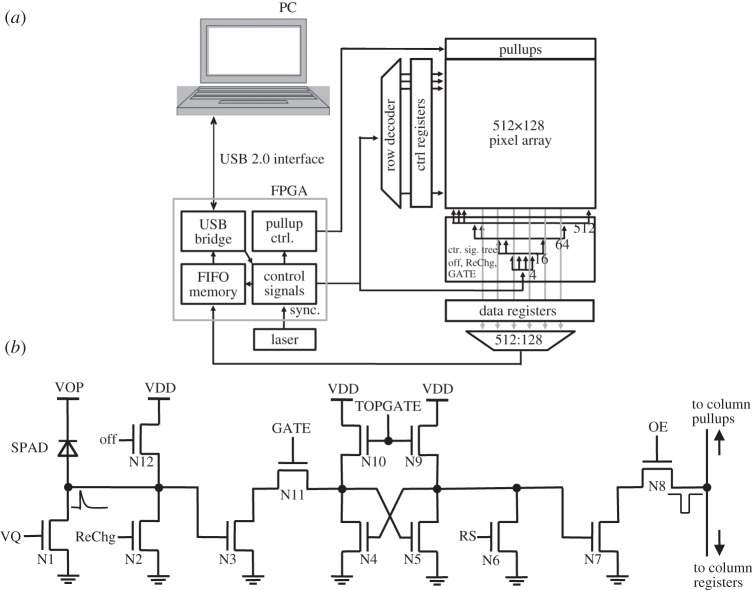
Block diagram of SwissSPAD (*a*); schematic diagram of the pixel with embedded one-bit counter and readout circuit (*b*). The counter is implemented as a static memory. The content of the counter is read out using a simple pulldown transistor and it may be set and reset using appropriate controls [68]. A detailed description of the pixel operation and of the symbols used in the schematics are given in the text.

In this design, time discrimination, photon counting and any additional functionality, including local storage, are performed on pixel, thus implementing full parallelism [68]. The pixel schematic, inspired by [44] and similar to [69] and [70], is shown in figure 31; it includes a 2.5 ns gating circuitry and it is implemented in all-NMOS style to minimize the pitch, achieving 24 μm with a fill factor of 5%. The SPAD’s *pn* junction is achieved between the *p*^+^ and *n*^−^ well layers; *p*^−^ guard rings are used to prevent PEB, whereas well sharing was not adopted to suppress electrical crosstalk. Transistor N1 is used to quench the SPAD, whereas the avalanche pulse at the cathode of the SPAD is applied via N3 to set the latch N4–N5–N9–N10, which, in turn, is reset by N6. The latch power consumption–speed trade-off is controlled by TOPGATE that can also turn the latch off completely. The latch drives N7 that is used as pulldown of the column line via selector N8, controlled by OE. The line voltage is sensed at the bottom of the column by a buffer and its value is saved in a one-bit register that is multiplexed to save I/O pads and power. The SPAD is gated by transistors N2, N11 and N12 using a classic off-and-recharge scheme [14]. First, the cathode voltage is raised from ground (where it is in idle state) to VDD via signal ‘off’ acting on N12. At this voltage, the SPAD bias is below breakdown, and thus it cannot be triggered by single photons. The SPAD is in that position until signal ReChg is asserted, thus bringing the SPAD again in Geiger mode of operation via N2. Note that Off and ReChg must not be overlapping to avoid a direct through current path that would increase power consumption by creating a direct path from VDD to ground. During the gating period signal, GATE must be asserted, so as to propagate potential pulses triggered by single photons.

**Figure 32.**
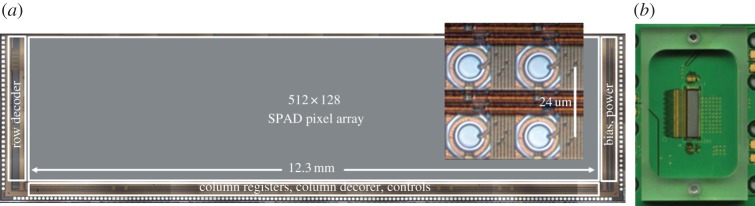
Photomicrograph of SwissSPAD, a 512×128 parallel-counting pixel array implemented in 0.35 μm CMOS technology (*a*); the inset shows a zoom of 4×4 pixels [68]. Printed circuit board bonded device (*b*). (Online version in colour.)

The chip micrograph is shown in figure 32, with a detail of the pixels and their column data readout interconnect and row-wise control lines. To construct images with multi-bit grey levels, a high-frequency readout was put in place capable of reading an entire one-bit frame in 5 μs, much in the same way as in [44]. Thanks to the speed of this architecture, moderate time-resolution techniques, such as fluorescence correlated spectroscopy, are possible on a much larger pixel scale than earlier attempts [71].

#### (iii) MEGAFRAME

**Figure 33.**
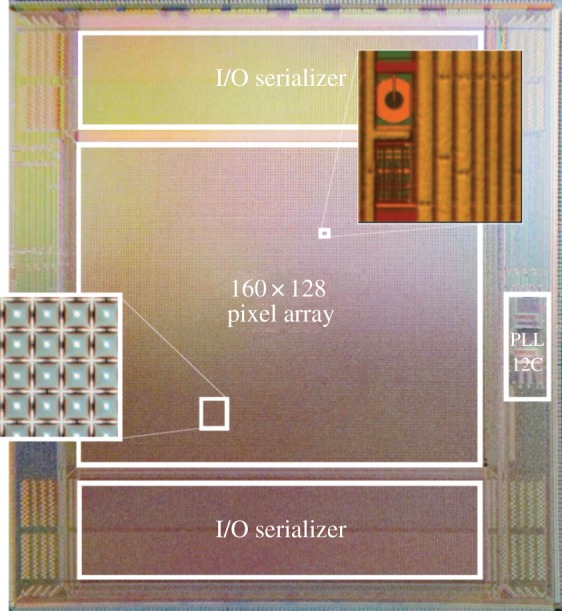
Photomicrograph of MEGAFRAME, a 160×128 pixel array, capable of performing one million TOA evaluations per pixel per second at 52 ps time resolution. In the insets, a pixel and 4×4 microlens array are visible. (Online version in colour.)

With the implementation of the first SPADs in 130 nm CMOS technologies [25,27] and 90 nm [30,31], it has been possible to integrate more functionality on pixel. The pixels of the array in the MEGAFRAME project for example comprise a multi-bit counter and a deep-subnanosecond resolution TDC [46–48]. One of the available implementations of the MEGAFRAME concept comprises an array of 32×32 pixels *each* of which is capable of performing TOA measurements with picosecond resolution and digital photon counting; it was conceived to operate both in TCSPC and TUPC modes. In TCSPC mode, the TDC in each pixel is enabled; it can determine and store the first of 10 TOA measurements in every frame of a length of a microsecond. In TUPC mode, the counter in each pixel is enabled; it can count up to 64 photon arrivals per microsecond. Figure 33 shows a photomicrograph of the implementation of MEGAFRAME reported in [72].

**Figure 34.**
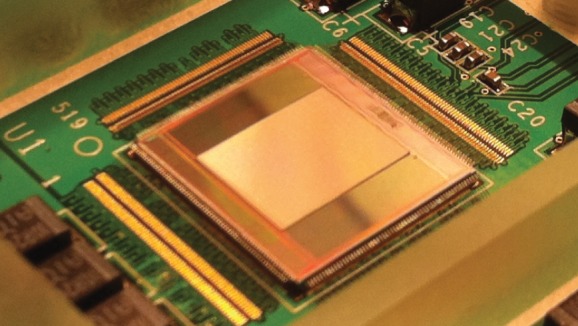
The MEGAFRAME chip mounted on a printed circuit board. The microlens array is visible in the centre of the picture. A full characterization of an identical microlens array is reported in [75]. (Online version in colour.)

The chip and its predecessors were demonstrated in a number of FLIM-related applications (e.g. [73,74]), to name a few. The design includes a phase-lock loop frequency synthesizer that generates the clock signals necessary to operate the TDCs. A I^2^C block also integrated in the chip manages the various modes of operation seamlessly. The overall chip and pixel architectures are described in more detail and fully characterized in [46–48,72,73]. Figure 34 shows the chip mounted on a printed circuit board with the microlens array deposited directly onto the SPAD array to reclaim a portion of the fill factor. A full characterization of an identical microlens array is reported in [75].

Other SPAD-based sensors focusing on FLIM were also proposed by other authors [76,77] with different formats and CMOS technologies.

Table 2 is a summary of the performance of four representative image sensors characterized by random access, event-driven readout and on-pixel TOA evaluation. The imagers were implemented in a variety of CMOS processes and thus a fair comparison is not possible. Because the first two architectures in table 2 do not include integrated TACs/TDCs, we report the timing uncertainty as it is evaluated externally, using a commercial TDC, whereas timing resolution and differential/integral nonlinearity are reported elsewhere. The overall pixel bandwidth refers to the maximum symbol rate that the image sensor can generate per pixel (irrespective of whether TOA is computed on- or off-chip). When TOA is computed off-chip, we assumed that processing speed is not limited by the TAC/TDC used but by intrinsic I/O speed. This is why the design of Niclass *et al.* [65] is penalized in the table with respect to the two previous designs, as the integrated TDCs are the bottleneck. In this design in fact, only one row can be operational at each time, whereas each four columns share a TDC. Thus, the overall TDC bandwidth of 10 MS s^−1^ must be divided by 4 times 128, to reach the reported value.

**Table 2.** Performance of CMOS SPAD imagers for three representative architectures.

	measurement	min.	typical	max.	unit
random access readout [22] architecture style (3)	format		32×32		—
	fill factor		1.1		%
	timing uncertainty or jitter (FWHM)		115		ps
	pixel pitch		58		μm
	overall pixel bandwidth		10		kS s^−1^
	count rate			10	kc s^−1^
	timing uniformity			1	%
	power dissipation			6	mW
event-driven readout [23,24] architecture style (2)	format		4×112		—
	fill factor		12.5		%
	timing uncertainty or jitter (FWHM)		80		ps
	pixel pitch		25		μm
	overall pixel bandwidth		223		kS s^−1^
	count rate			223	kc s^−1^
	timing uniformity		—		%
	power dissipation			7	mW
in-column TOA evaluation [65] architecture style (2)	format		128×128		—
	fill factor		6		%
	timing resolution (LSB)		97.68	70	ps
	differential/integral nonlinearity (DNL/INL)			0.08/1.89	LSB
	pixel pitch		25		μm
	overall pixel bandwidth		19.5	2500	kS s^−1^
	count rate		19.5	2500	kc s^−1^
	TOA uniformity		—		LSB
	power dissipation		33	150	mW
on-pixel TOA evaluation [72] architecture style (1)	format		160×128		—
	fill factor		1		%
	timing resolution (LSB)		55	119	ps
	differential/integral nonlinearity (DNL/INL)		±0.3/±2		LSB
	pixel pitch		50		μm
	overall pixel bandwidth		50		kS s^−1^
	count rate			10 000	kc s^−1^
	TOA uniformity		0.5		LSB
	power dissipation		550		mW

In the design of Gersbach *et al.* [46], a bandwidth of 1 MS s^−1^ in TCSPC mode can be achieved, whereas a much higher count rate is possible, thanks to an on-pixel six-bit counter. Thus, the maximum count rate is limited by the dead time of 100 ns. The timing uniformity, wherever measured, is expressed in % or LSB depending on the presence of TOA evaluation on-chip.

## 5. Conclusion

In this paper, we have reviewed single-photon image sensors based on SPAD technology, covering SPAD fundamentals, characterization of SPAD-based imagers and architectures. The most important architectures available today are presented in the context of SPAD image sensors fabricated in CMOS processes. For the architecture selection, it is shown how critical the target application is, whereas proper circuit design techniques can be used to reduce the impact of supply and substrate noise. Deep-submicrometre CMOS SPAD imagers are possible today with a performance comparable to that of state-of-the-art single-pixel detectors implemented in dedicated technologies, but with a massive number of pixels operating simultaneously. The applications are endless, from biomedicine to chemistry, from engineering to entertainment.

**Figure 35.**
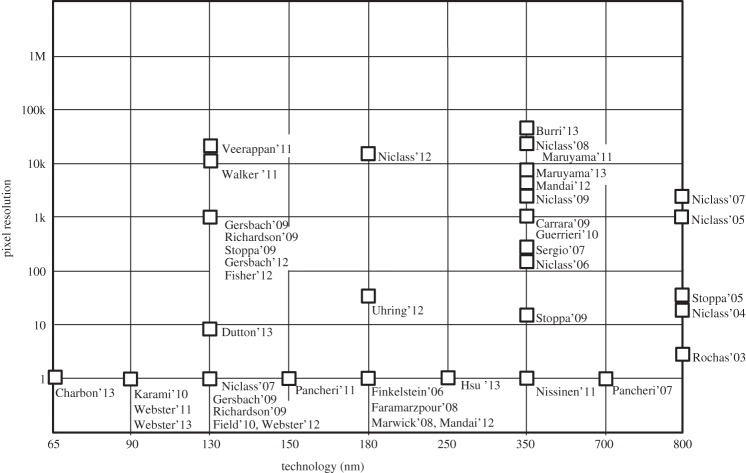
SPAD image sensor development landscape based on articles published in the period 2003–2013. The pixel resolution relates to the size of the SPAD array; each technology node is represented by its feature size.

The landscape in single-photon imaging has rapidly developed in recent years with the emergence of SPADs and SPAD-based imagers ranging from a few pixels to large arrays and from low functionality to large degrees of complexity both at pixel and system level. Figure 35 shows a graphical impression of this landscape as a function of pixel count for the various CMOS technologies published in the 2003–2013 decade.
